# Pathway Thermodynamics Highlights Kinetic Obstacles in Central Metabolism

**DOI:** 10.1371/journal.pcbi.1003483

**Published:** 2014-02-20

**Authors:** Elad Noor, Arren Bar-Even, Avi Flamholz, Ed Reznik, Wolfram Liebermeister, Ron Milo

**Affiliations:** 1 Department of Plant Sciences, The Weizmann Institute of Science, Rehovot, Israel; 2 Department of Molecular and Cellular Biology, University of California, Berkely, Berkely, California, United States of America; 3 Computational Biology Program, Memorial Sloan-Kettering Cancer Center, New York, New York, United States of America; 4 Institut für Biochemie, Charité-Universitätsmedizin Berlin, Berlin, Germany; University of Michigan, United States of America

## Abstract

In metabolism research, thermodynamics is usually used to determine the directionality of a reaction or the feasibility of a pathway. However, the relationship between thermodynamic potentials and fluxes is not limited to questions of directionality: thermodynamics also affects the kinetics of reactions through the flux-force relationship, which states that the logarithm of the ratio between the forward and reverse fluxes is directly proportional to the change in Gibbs energy due to a reaction (Δ_r_G′). Accordingly, if an enzyme catalyzes a reaction with a Δ_r_G′ of -5.7 kJ/mol then the forward flux will be roughly ten times the reverse flux. As Δ_r_G′ approaches equilibrium (Δ_r_G′ = 0 kJ/mol), exponentially more enzyme counterproductively catalyzes the reverse reaction, reducing the net rate at which the reaction proceeds. Thus, the enzyme level required to achieve a given flux increases dramatically near equilibrium. Here, we develop a framework for quantifying the degree to which pathways suffer these thermodynamic limitations on flux. For each pathway, we calculate a single thermodynamically-derived metric (the Max-min Driving Force, MDF), which enables objective ranking of pathways by the degree to which their flux is constrained by low thermodynamic driving force. Our framework accounts for the effect of pH, ionic strength and metabolite concentration ranges and allows us to quantify how alterations to the pathway structure affect the pathway's thermodynamics. Applying this methodology to pathways of central metabolism sheds light on some of their features, including metabolic bypasses (*e.g*., fermentation pathways bypassing substrate-level phosphorylation), substrate channeling (*e.g*., of oxaloacetate from malate dehydrogenase to citrate synthase), and use of alternative cofactors (*e.g*., quinone as an electron acceptor instead of NAD). The methods presented here place another arrow in metabolic engineers' quiver, providing a simple means of evaluating the thermodynamic and kinetic quality of different pathway chemistries that produce the same molecules.

## Author Summary

Given data about enzyme kinetics and reaction thermodynamics, traditional metabolic control analysis (MCA) can pinpoint the enzymes whose expression will have the largest effect on steady-state flux through the pathway. These analyses can aid experimentalists in tuning enzyme expression levels along a metabolic pathway. In this work, we offer a framework that is complementary to MCA. Rather than focusing on the relationship between enzyme levels and pathway flux, we examine a pathway's stoichiometry and thermodynamics and ask whether it is likely to support high flux in cellular conditions. Our framework calculates a single thermodynamically-derived metric (the MDF) for each pathway, which is convenient for selecting the promising pathways from a large collection. This approach has several advantages. First, enzyme kinetic properties are laborious to measure and differ between organisms and isozymes, but no kinetic data is required to calculate the MDF. Second, as our framework accounts for pH, ionic strength and allowed concentration ranges, it is simple to model the effect of these parameters on the MDF. Finally, as it can be difficult to control the exact expression level of enzymes within cells, the MDF helps identify alternative pathways that are less sensitive to the levels of their constituent enzymes.

## Introduction

A primary scientific goal of metabolic research is to develop an understanding of the evolutionary, chemical and physical forces that shape the structure of cellular metabolism. Specifically, to what extent are present-day metabolic pathways the result of evolutionary optimization rather than fossilized accidents? In recent years various aspects of central metabolism have been explained on the basis of specific selection pressures and constraints imposed during evolution [1], [2], [3], [4], [5], [6], [7], [8], [9], [10], [11], [12], [13]. Among the various constraints that shape the structure of metabolic pathways, thermodynamics features prominently, linking fundamental physical properties to pathway architecture [1], [9], [10], [11], [14], [15], [16]. Thermodynamic profiling also plays a central role in synthetic pathway design by identifying the most promising candidate pathways and discarding infeasible ones [14], [17], [18], [19], [20], [21].

**Figure 1:**
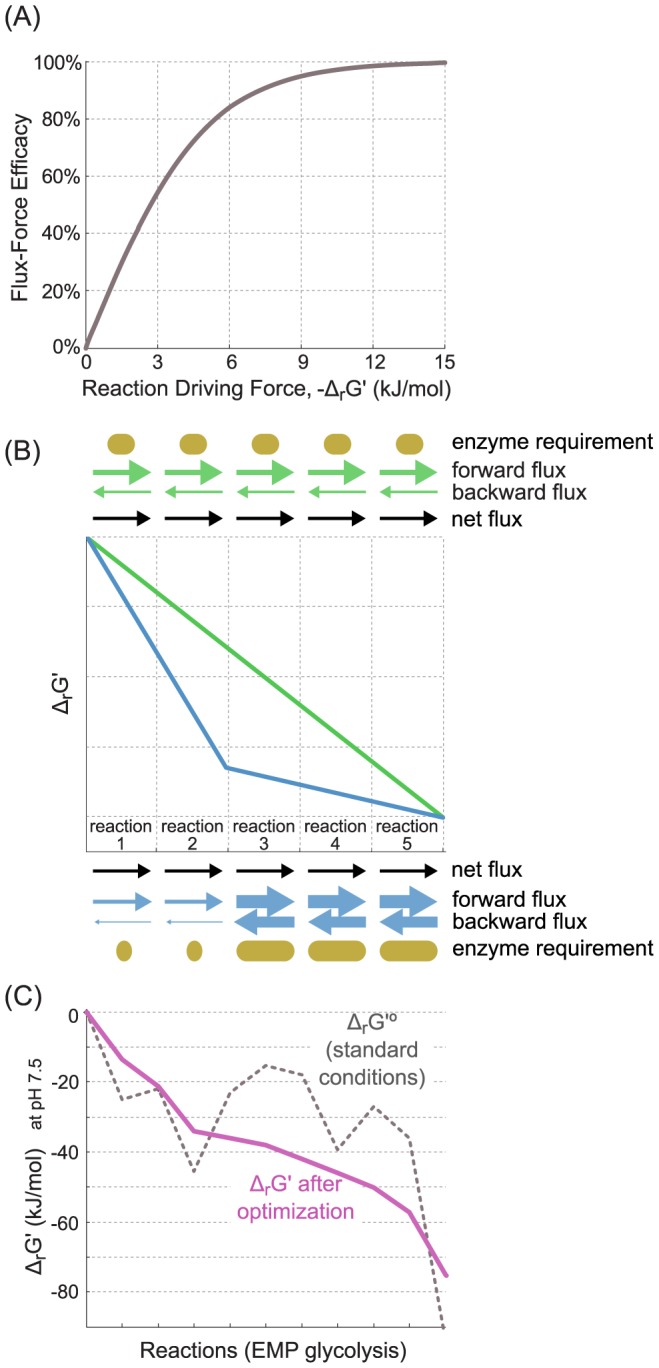
The Flux-Force Efficacy and Minimum Optimized Driving-Force (MDF). (A) A functional relationship between the reaction driving force (−Δ_r_G′) and its Flux-Force Efficacy, as described in detail in the Methods section. (B) Schematic comparison between two pathways. Each pathway starts and ends with the same compounds, employs five enzymes and carries the same net flux. The kinetic parameters of all enzymes in both pathways, as well as enzyme and metabolite concentrations, are assumed to be identical. (C) Energetic profile of Embden-Meyerhof-Parnas glycolysis. Dashed black line corresponds to Δ_r_G′^o^ values (metabolite concentrations of 1 M) of pathway reactions at pH 7.5. Red line corresponds to Δ_r_G′ values of pathway reactions after an optimization procedure that maximizes the driving force of the reactions having the lowest driving forces, as described in the Methods section.

Thermodynamic analysis is typically applied to determine whether a reaction direction or pathway is feasible in physiological conditions [9], [22]. Although not widely appreciated, thermodynamic potentials also constrain the kinetics of biochemical reactions and pathways [13], [23], [24], [25]. Specifically, the Gibbs energy dissipated by a reaction, Δ_r_G′, affects the net reaction rate through the flux-force relationship [23]: Δ_r_G′ = −RTln(J^+^/J^−^), R being the gas constant, T the temperature, J^+^ the forward flux and J^−^ the backward flux. Consequently, an enzyme catalyzing a reaction that is far from equilibrium (Δ_r_G′<<0) carries almost no backwards flux (Figure 1a) while an enzyme catalyzing a near-equilibrium reaction (Δ_r_G′∼0) “wastes” many enzyme units catalyzing substantial flux through the reverse reaction. According to the flux-force relationship, as a reaction shifts towards equilibrium we would see an exponential increase in the number of enzyme units required to catalyze a single unit of flux. For example, a Δ_r_G′ of −7.3 kJ/mol implies that about 5% of the enzymatic flux is in the reverse direction. Alternatively, a rather close-to-equilibrium Δ_r_G′ of −1 kJ/mol implies that about 40% of the enzymatic flux is in the reverse direction and the reaction rate is only ∼20% of the rate that would be achieved if all enzyme units catalyzed the forward reaction.

Metabolic Control Analysis (MCA) is commonly used to describe the control that enzymes exert on metabolic fluxes [26], [27], [28], [29], [30]. This methodology starts with a given steady-state and mathematically describes how changes in enzyme abundance affect the pathway flux. Application of MCA requires enzyme kinetic properties which are laborious to measure and differ between organisms and isozymes. Here, we describe a complementary approach that requires no kinetic data and is not dependent on a particular initial steady state. We aim to identify pathways that, due to their thermodynamics, likely require higher enzyme levels to catalyze a unit of flux. Further, we pinpoint the particular pathway reactions responsible for these thermodynamic limitations. The flux-force relationship is instrumental in these analyses as it expresses a relationship between the Gibbs energy dissipated during a reaction (Δ_r_G′) and the amount of enzyme required to sustain a particular flux through that reaction. Therefore, the protein burden imposed by a pathway is directly related to the thermodynamic landscape of that pathway.

We develop a quantitative framework to analyze the thermodynamic landscape of metabolic pathways. Our framework identifies those reactions within a pathway whose rates are constrained by low thermodynamic driving force. These enzymes will constrain the activity of the pathway unless they are present at high concentrations or are much faster-than-average catalysts [31]. Using this methodology it is straightforward to compare different pathways that achieve similar metabolic goals (unlike MCA, which assumes a particular steady-state for each pathway, making comparison difficult). To demonstrate our method, we apply it to pathways of central metabolism, including fermentation pathways (e.g., Embden-Meyerhof-Parnas (EMP) glycolysis) and oxidative pathways (e.g., TCA cycle). We compare various alternative pathways by their thermodynamic landscapes and identify the reactions supported by a low thermodynamic driving force and, hence, requiring a high enzyme expression level.

## Methods

### Thermodynamic parameters

We used the Component Contribution method [32] for estimating the standard Gibbs energies of reactions, Δ_r_G′^o^ (reactant concentrations of 1 M). This method produces a consistent set of Δ_r_G′^o^ values by integrating three sources of information in decreasing priority: (1) ∼200 Gibbs energies of formation (Δ_f_G^o^) collected and published by Alberty [33], [34]; (2) apparent equilibrium constants (K′) available in the NIST database of enzyme-catalyzed reactions [35], [36]; and (3) the pseudoisomeric group contribution method as described in detail in ref. [22]. All Δ_r_G′^o^ values were transformed to a pH of 7.5 and an ionic strength of 0.2 M [22], representing typical cellular conditions [37]. We use these conditions in all analyses presented in this paper, unless stated otherwise.

### Driving force and pathway feasibility

The Gibbs energy dissipated by a reaction can be calculated from the standard Gibbs energy of the reaction (*Δ_r_G′^o^*) and the reactant concentrations. It is given by *Δ_r_G′ = Δ_r_G′^o^+RT·ln(Q)*, where *Q* is the reaction quotient (also known as the mass action ratio). Because of its more intuitive sign we will often refer to a reaction's *Driving Force*, defined as *−Δ_r_G′* [38].

When analyzing pathways containing multiple reactions, it is convenient to use matrix notation [39]. We define **S** as the stoichiometric matrix, with rows corresponding to compounds and columns to reactions; *i.e*., S_ij_ is the stoichiometric coefficient of compound *i* in reaction *j* (positive for products and negative for substrates). ***G^o^*** *denotes* a column vector of reaction energies, *i.e*., *G^o^_j_* is the standard change in Gibbs energy (*Δ_r_G′^o^*) of reaction *j*. Finally, let ***x*** be the column vector of the log-concentrations, so that *x_i_* is the natural logarithm of the concentration of compound *i* in molar units. Then the vector of reaction driving forces (**−** ***Δ_r_G′***) is given by −*(* ***G^o^*** *+RT*·**S** ^T^·***x*** *)*.

We use a convention where all reactions in **S** are written such that the forward direction is the direction of the net flux in the pathway, and the stoichiometric coefficients represent the actual molecularities in the enzyme's reaction center [38] (*i.e*., the number of reactant molecular entities that are involved in the ‘microscopic chemical event’ constituting an elementary reaction). This convention obviates the situation existing in more general stoichiometric models [40], where scaling the flux of a reaction by a scalar and dividing the stoichiometric coefficients of that reaction by the same factor results in an equivalent system. Using the actual molecularities is vital for our analysis, as the flux-force relationship cannot accept an arbitrary definition of stoichiometry.

Considering ***G^o^*** to be constant and allowing ***x*** to vary, a pathway is feasible if and only if there is at least one solution to the linear system defined by the constraints ln(*C_min_*)≤***x***≤ln(*C_max_*) and **−** ***Δ_r_G′*** *>0*; *i.e*., there must exist a set of metabolite concentrations within the predefined range (*C_min_* to *C_max_*) such that all reactions have a positive driving force. The concentration bounds (*C_min_* and *C_max_*) may be the same for all compounds or can be defined individually.

Even if a pathway is composed of thermodynamically-feasible reactions, the pathway may be thermodynamically infeasible as a whole. That is, no solution for ***x*** exists within the chosen concentration range such that **−** ***Δ_r_G′*** *>0* and so the pathway reactions cannot all be made feasible simultaneously [41].

### Flux-Force Efficacy and Max-min Driving Force (MDF)

As described in the Introduction, the driving force of a reaction constrains its rate, with near-equilibrium reactions requiring exponentially more enzyme to sustain the same rate as reactions far from equilibrium. We define the *Flux-Force Efficacy* – a unitless measure between 0 and 1 – as the ratio between the net flux (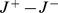) and the total flux (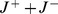), which – according to the flux-force relationship – is related to the change in Gibbs free energy by 
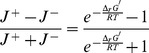
. Hence, the higher the driving force of a reaction, the higher its *Flux-Force Efficacy*.

Because of this interdependence between thermodynamic potentials and flux, pathways operating near equilibrium will incur a kinetic penalty due to backwards flux. We therefore attempt to quantify a pathway's tendency to operate near-equilibrium. We seek a set of reactant concentrations that will maximize the driving forces of all reactions in the pathway. To achieve this, we use the minimum over all reaction driving forces (−*Δ_r_G′* values of pathway reactions) as an optimization goal and maximize it – within metabolite concentration bounds – using linear programming. This can be formalized as a linear problem:
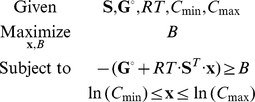
where *B* represents a tight lower bound (*i.e*., the minimum) on the driving force of all reactions. Maximizing *B* yields a solution where all reactions are as far from equilibrium as possible given the defined concentration ranges. The maximal value of *B* is denoted as the ***M*** *ax-min* ***D*** *riving-* ***F*** *orce* (MDF) of the pathway and is measured in units of kJ/mol [42].

If a pathway has an MDF of 7.3 kJ/mol then a set of metabolite concentrations exists, within the pre-defined concentration range, such that all pathway reactions dissipate at least 7.3 kJ/mol. A Δ_r_G′ = −7.3 kJ/mol corresponds to a J^+^/J^−^ ratio of exp(7.3/RT) = 19, which in turn suggest that 95% of the overall flux is in the forward direction and 95/19 = 5% is in the backward direction. Therefore, the *Flux-Force Efficacy* is 95−5 = 90% (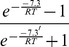 = 0.9). Since the MDF is a tight bound, it is impossible to find a set of concentrations within the specified range for which all reaction driving forces are larger than, say, 7.4 kJ/mol. Notably, the MDF solution is also equivalent to minimizing the *total* enzyme mass in a linear pathway, assuming that all enzymes have the same specific activities (see Text S1 for a mathematical proof).

### Reaction and Metabolite Shadow Prices

In order to quantify the extent to which a reaction or a metabolite affects the value of the MDF, we use the concept of shadow prices [42], [43]. Every primal linear optimization problem has a complementary dual problem. The variables of the dual problem – called shadow prices – quantify how much the value of the primal objective – *i.e*., the MDF – will increase when a single constraint is relaxed by a unit amount. There are three types of constraints in the MDF linear problem: the lower bound (*B*) of the reaction driving forces (−***G^o^***−*RT*· **S** ^T^ · ***x***≥*B*), the upper bound of metabolite concentrations (***x***≤ln(*C_max_*)), and the lower bound of metabolite concentrations (ln(*C_min_*)≤***x***). We therefore define the *Reaction Shadow Price* as the shadow price associated with the constraint on the driving force of that reaction, representing how much a change in ***G^o^*** will affect the MDF. The *Metabolite Shadow Price* is the maximum of the absolute values of the two shadow prices associated with the constraints (lower and upper bound) on that metabolite's concentration.

The shadow prices are the solution for the variables of the dual problem **w**, **u_max_**, **u_min_**:
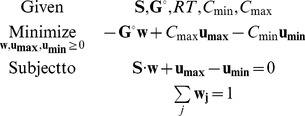



According to the definitions above, **w** are the *reaction* shadow prices and max(|**u_max_** *|*, |**u_min_** *|*) are the *metabolite* shadow prices. See Text S1 for a full derivation of the dual problem.

The shadow prices represent a scaling between a change in the constraint and the resulting change in the MDF. For example, a reaction shadow price of 0.25 indicates that a 4 kJ/mol decrease in Δ_r_G′^o^ would increase the pathway MDF by 1 kJ/mol (assuming that this reaction still limits the pathway MDF). Similarly, a metabolite shadow price of 0.5, associated with the upper bound constraint, implies that raising the upper bound concentration of this metabolite by 10 fold will result in the MDF increasing by 0.5*RTln(10)≈3 kJ/mol. Shadow prices are 0 for reactions whose Δ_r_G′^o^ does not constrain the MDF, and likewise for metabolites whose concentrations do not constrain the MDF.

### Metabolite concentration range

Throughout our analyses we used metabolite concentration bounds characteristic of cellular physiology, a lower bound *C_min_* = 1 µM and an upper bound *C_max_* = 10 mM [9], [44], [45]. An exception was made for cofactors, whose concentrations were fixed to those characteristic of *E. coli*'s cytoplasm. Cofactors participate in tens or even hundreds of reactions and so their concentrations are considerably more constrained by the endogenous metabolic network than common reaction intermediates [46]. Fixing the concentrations of cofactors allows us to encode these constraints imposed by the wider metabolic network on individual pathways. Wherever possible, we constrained the cofactor ratios rather than their absolute concentrations, since the ratios are more conserved in many cases. The co-factor constraints we use are as follows: [ATP]/[ADP] = 10 [45], [47], [48], [49], [ADP]/[AMP] = 1 [45], [49], [NADH]/[NAD^+^] = 0.1 [45], [50], [NADPH]/[NADP^+^] = 10 [45], [51], [Ferredoxin_reduced_]/[Ferredoxin_oxidized_] = 1 (corresponds to a reduction potential of −400 mV [52]), [orthophosphate] = 10 mM [53], , [pyrophosphate] = 1 mM [56], [57], [CoA] = 1 mM [45], [CO_2_(aq)] = 10 µM (ambient conditions).

### Connection between thermodynamic driving forces and Metabolic Control Analysis

Enzyme abundances control the steady-state fluxes within a pathway [26], [27], [28], [29], [30]. When an enzyme is upregulated, the rate of the reaction it catalyzes increases instantaneously, but the rates of other reactions in the pathway do not change at first. This state, however, cannot be maintained for long as the concentration of the enzyme's substrates decrease, while its products accumulate. Therefore, other reactions in the pathway are affected, and eventually, after the system settles in a new steady state, all fluxes might be altered. The term “control” describes such indirect, global effects.

Potentially, all enzymes exert control on all fluxes within the pathway, but to different extents. Metabolic Control Analysis describes this control mathematically: if we consider small changes to a given steady-state, the effect of an enzyme's abundance on the pathway flux, can be quantified by the scaled flux control coefficient [26], [58]:
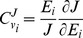
where *J* is the steady-state flux, *v_i_* is the rate of reaction *i* and *E_i_* is the abundance of the enzyme catalyzing reaction *i*. In the general case, control coefficients depend on the pathway structure, enzymatic parameters, and allosteric regulation. Since all control coefficients for a flux *J* always sum to 1 [58], [59], control can only be redistributed among the different pathway enzymes.

The flux control coefficients are related to the thermodynamic driving forces, as derived in Text S1 and was shown in ref. [26]. This relationship is easily derived for linear pathways whose enzymes follow the reversible Michaelis-Menten rate laws [26], [60], [61]. In Text S1, we derive the relationship for two specific cases: (1) all enzymes are completely substrate (but not product) saturated. Importantly, because all enzymes are essentially reversible, the rates of all reactions are sensitive to the concentrations of the substrates and products, even if all enzymes are substrate saturated. A full analysis of this case is given ref. [61]; (2) the substrates and products of all enzymes are well below their K_M_ (enzymes operate in the linear regime). We show that in both cases the control coefficients are completely determined by reaction driving forces, such that the two are always correlated: the higher the driving force of a reaction, the higher the control it exerts on the pathway flux. Specifically, reactions with low driving forces have very low control coefficients.

For the first case – all enzymes are substrate-saturated – we find that 
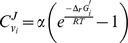
.

For the second case – when all enzymes are substrate and product sub-saturated – we find that:


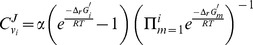
, in agreement with the derivation of [26].

In both cases the scaling factor α is identical for all reactions and is determined by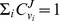.

### Software

The software for all the analyses presented in this paper is open source (MIT license) and stored in an online repository (https://code.google.com/p/milo-lab/). We use IBM's ILOG CPLEX Optimization Studio 12.5 to solve the MDF primal and dual problems. The code which predicts the standard Gibbs energies [32] also depends on OpenBabel (http://openbabel.org) [62], Calculator Plugins from Marvin (version 5.10.1) by ChemAxon, and the KEGG database (http://www.genome.jp/kegg/) [63].

## Results

### The Flux-Force Efficacy and the Max-min Driving-Force (MDF)

The kinetics of a reaction can be linked to three main factors: (1) maximal velocities and saturation levels, related to the enzyme kinetic parameters and to the concentrations of substrates and products; (2) enzyme abundances and (3) reverse flux though reactions [13]. Thermodynamics determines this last factor through the flux-force relationship: the reaction driving force (equivalent to the minus of the reaction change in Gibbs energy, *i.e.,* −Δ_r_G′) equals RTln(*J^+^*/*J^−^*), as presented above. For low enough driving forces (roughly 3 kJ/mol or less) the effect of Δ_r_G′ on the reaction flux is similar to that of k_cat_ (or V_MAX_) in the sense that a fold change in either of them will change the reaction rate by the same fold change. The dependence of the reaction rate on −Δ_r_G′ decreases as −Δ_r_G′ increases above ∼3 kJ/mol. When −Δ_r_G′ exceeds 10 kJ/mol, the reaction rate is effectively insensitive to thermodynamic effects, reflecting the fact that there is negligible flux in the reverse direction.

To address the effect of driving force on reaction flux more systematically, we define the Flux-Force Efficacy as the ratio between reaction net flux and total flux, 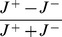 (Methods). The Flux-Force Efficacy can be interpreted as the ratio between the actual reaction rate and the rate expected if backward flux was insignificant (assuming maximal velocities, saturation levels and enzyme concentrations are kept constant). Figure 1A shows how this ratio scales with the reaction driving force.

We use the term “efficacy”, instead of “efficiency”, to distinguish between Flux-Force Efficacy and thermodynamic efficiency, as the two are antagonistic. For example, a reaction operating close to equilibrium is often considered to be thermodynamically efficient since it dissipates almost no Gibbs energy. Yet, such a reaction is characterized by a low Flux-Force Efficacy as *J^+^*≈*J^−^*. In contrast, a reaction with a high Flux-Force Efficacy, characterized by *J^+^*>>*J^−^*, is thermodynamically inefficient, dissipating a considerable amount of Gibbs energy.

For a fixed enzyme level, a high Flux-Force Efficacy implies a high net reaction rate, as backward flux is negligible. On the other hand, a low Flux-Force Efficacy indicates considerable backward flux, leading to a decreased reaction rate. In a complementary manner, we can use this relationship to estimate the amount of enzyme required to sustain a particular flux through the reaction [13]. Figure 1B demonstrates this effect schematically using the energetic profiles of two putative pathways. Both pathways start and end with the same compounds, employ five enzymes and carry the same net flux. The kinetic parameters of all enzymes in both pathways, as well as enzyme and metabolite concentrations, are assumed to be identical. All reactions in the green pathway have the same, moderate driving force, which translates to a small backward flux. Hence, a small amount of each enzyme suffices. In the blue pathway, the driving force of the first two reactions is large while the last three reactions are near equilibrium. These final three reactions, therefore, require a lot more catalyst in order to sustain the same flux as the first two reactions in the blue pathway.

When analyzing an entire pathway, it is essential to consider the interplay between the driving forces of all participating reactions: varying the concentration of a given metabolite can modulate the driving force of multiple reactions. We developed a method for finding metabolite concentrations – within an allowed range, see Methods – that maximize the driving force, and hence the Flux-Force Efficacy, of pathway reactions. Specifically, our computational tool uses the minimum over all reaction driving forces as an optimization function and maximizes it (Methods). This minimum, representing the smallest driving force among all pathway reactions, is defined as the **M**ax-min **D**riving-**F**orce (MDF).

Figure 1C illustrates the application of the optimization approach to EMP glycolysis. The grey dashed line represents the Δ_r_G′^o^ values of the different pathway reactions, while the magenta line represents the Δ_r_G′ values for each of the reactions after optimizing reactant concentrations to maximize the MDF. After this optimization, all reactions in the pathway have a positive driving force (*i.e*., a negative slope) and so it is clear that the EMP pathway is thermodynamically feasible.

Presuming the Δ_r_G′^o^ values used are accurate and that our concentration bounds reflect cellular concentrations, pathways are thermodynamically feasible if and only if they have a positive MDF. Moreover, the value of the MDF indicates the degree to which a pathway is expected to be kinetically constrained by backward flux. A pathway with a high MDF can achieve a steady-state with very low backward flux as all of its constituent reactions can achieve high driving forces simultaneously. On the other hand, a pathway characterized by a low MDF contains reactions that are expected to have low driving force in physiological conditions. Due to the flux-force relationship, these reactions must either sustain low flux or be catalyzed by an abundant enzyme. For example, the green pathway shown in Figure 1B operates at high MDF which results in a high pathway flux and/or a low enzyme requirement. On the other hand, the blue pathway operates at low MDF, containing near-equilibrium reactions which reduce pathway flux and/or require higher enzyme levels.

The shadow prices determine whether a specific reaction or metabolite constrains the pathway MDF, as described in detail in the Methods section. A decrease in the Δ_r_G′^o^ value of a reaction with a positive shadow price would lead to an increase in the MDF. Similarly, if the concentration of a metabolite with a positive shadow price is permitted to violate the allowed concentration range (becomes too high or too low), the MDF increases (Methods).

According to our model, enzymes catalyzing reactions with positive shadow prices are expected to be present at higher concentrations or have higher-than-average k_cat_ values. While it is tempting to test this hypothesis systematically, it is, unfortunately, very challenging using available experimental data. Specifically, the MDF analysis requires that the magnitudes of all fluxes are precisely determined (taking into account that the flux through enzymes participating in the same pathway might differ due to an overlap with other metabolic routes). However, even for the relatively simple case of *E. coli*'s central metabolism, flux and metabolite concentration measurements from different groups vary significantly, even when performed under similar conditions (*e.g*., [64], [65]). In addition, proteomic measurements related to lowly-expressed proteins are still quite noisy. Finally, most kinetic parameters reported in the literature were measured *in vitro*, which can differ considerably from those experienced *in vivo* [66], [67]. These issues limit our ability to perform a comprehensive systematic analysis of the relationship between the thermodynamic parameters, the measured k_cat_ values and enzyme levels. Our current contribution suggests specific predictions to be tested when the needed experimental technologies mature.

In the sections below we demonstrate our methodology by applying it to well-known central metabolic pathways. Our analysis, although not systematic, provides several examples of thermodynamic properties affecting pathway flux and suggests thermodynamic based explanations for key biochemical phenomena.

### Malate dehydrogenase constrains the Max-min Driving-Force of the TCA pathway

**Figure 2:**
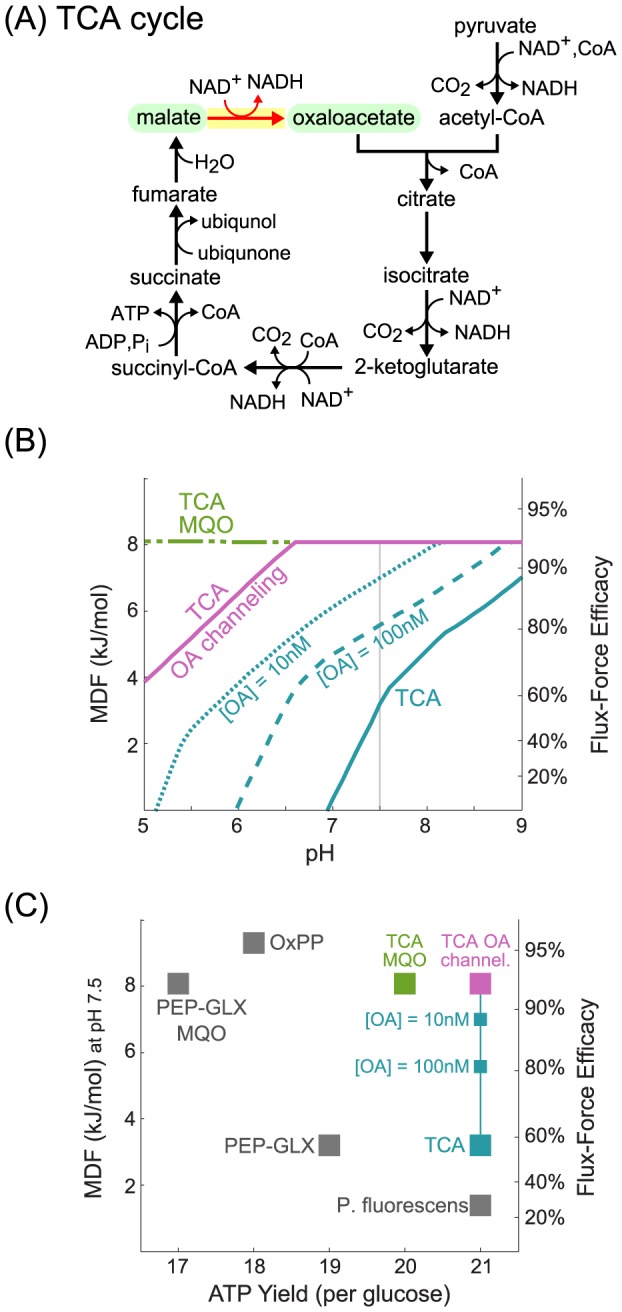
MDF analysis of oxidative pathways. (A) Structure of the TCA cycle. The reaction marked in red is the only one with a positive shadow price at pH 7.5. Non-cofactor metabolites shaded in green show positive shadow prices. (B) MDF as function of pH, as calculated for the TCA cycle and several of its similar variants. Solid cyan line: default metabolite concentration range used throughout this study (1 µM–10 mM). Dashed and dotted cyan lines: oxaloacetate concentration (marked as ‘OA’) is allowed to attain lower values, 100 nM and 10 nM, respectively. Solid magenta line: oxaloacetate is channeled (‘channeling’) between malate dehydrogenase and citrate synthase. Semi-dashed green line: quinone (‘MQO’) serves as the electron acceptor in malate oxidation, instead of NAD. The Flux-Force Efficacy axis, on the right, refers to the reactions that dissipate the smallest amount of Gibbs energy, and hence equal to the pathway MDF. The light grey line marks the values corresponding to pH 7.5, the pH used in (C). (C) The MDF and ATP yield per glucose of the different oxidative pathways. ‘PEP-GLX’ corresponds to the PEP-Glyoxylate pathway, which was found to operate in *E. coli* under glucose starvation [64]. ‘*P. fluorescens*’ corresponds to the pathway used by *Pseudomonas fluorescens* under conditions of aluminum toxicity [81]. ‘OxPP’ corresponds to the oxidative pentose phosphate cycle, which can be used to fully oxidize sugars into CO_2_, providing NADPH for cellular activity. Reducing power was assumed to be converted to ATP via oxidative phosphorylation, where NADH or a pair of reduced ferredoxins give rise to 1.5 ATP molecules and reduced ubiquinone produces one ATP molecule. The structures of all pathways are given in Figure S1.

In most organisms, the TCA cycle is the pathway responsible for the catabolic oxidation of organic compounds to CO_2_ (Figure 2A). Figure 2B presents the MDF of the TCA cycle (solid blue line) as a function of pH. We chose to vary pH, rather than other factors that affect the MDF, because cellular pH can differ considerably between organisms [37] and because the thermodynamics of many biochemical reactions producing or consuming protons is greatly affected by changes in pH. Figure 2B shows that the TCA cycle has a low MDF. In fact, it seems infeasible at pH≤7, which contradicts the observation that numerous organisms operate the TCA cycle at low cytosolic pH values [37]. To understand this puzzling finding, we asked which reaction(s) are responsible for constraining the pathway's MDF – *i.e*., which reactions have a positive shadow price. We find that, at non-alkaline conditions, the only reaction with a positive shadow price is malate dehydrogenase. The oxidation of malate to oxaloacetate using NAD as an electron acceptor (marked in red in Figure 2A) is characterized by a large positive Δ_r_G′^o^ (>30 kJ/mol at pH≤7). How, then, are cells able to sustain high flux through the TCA cycle? The MDF framework enables us to suggest solutions for this apparent paradox.

First, a high turnover number can compensate for operating at a low MDF: if the maximal activity of an enzyme is high enough, it will be able to operate sufficiently fast even at a low Flux-Force Efficacy. For example, an enzyme having a k_cat_ of 100 s^−1^ and catalyzing a reaction with a driving force of only 0.3 kJ/mol (Flux-Force Efficacy ∼6%) is equivalent to an enzyme having a k_cat_ of 10 s^−1^ (the average k_cat_ [31]) but catalyzing a reaction with a driving of 3 kJ/mol (Flux-Force Efficacy >50%). Thus, the high turnover number of malate dehydrogenase (well above 1000 s^−1^ [31]) might have evolved to compensate for its low driving force. However, this compensation effect does not answer how the cycle can carry flux at pH≤7, when malate oxidation is expected to become infeasible.

Another possible explanation is that the concentration of oxaloacetate – having a positive shadow price (Figure 2A) – is lower than 1 µM, the lower-bound concentration assumed in our analysis. As oxaloacetate is an unstable compound [68], it is tempting to suggest that it is indeed found at a sub-micromolar concentration *in-vivo*. As shown in Figure 2B, allowing oxaloacetate concentrations beneath 1 µM increases the pathway's MDF and the pH range in which it is thermodynamically feasible. However, keeping the concentration of oxaloacetate so low might be deleterious, as it would limit the rate of reactions which utilize this metabolite, *e.g*., citrate synthase, aspartate transaminase and PEP carboxykinase. In fact, the relatively high affinity of citrate synthase towards oxaloacetate – K_M_ being on the order of 1 µM [31], [69] – can be interpreted as representing an adaptation towards a low oxaloacetate concentration.

A further possibility, also supported by experimental studies, is that oxaloacetate is channeled between malate dehydrogenase and citrate synthase [70], [71], [72], [73]. If channeling indeed takes place, the cellular concentration of oxaloacetate can be extremely low without compromising the rate of the enzymes utilizing it. From a thermodynamic point of view, malate dehydrogenase and citrate synthase can then be treated as a single reaction [9], [74]. This unified reaction does not represent any thermodynamic difficulty as its Δ_r_G′^o^ is lower than −20 kJ/mol. As shown in Figure 2B, such a scenario increases the pathway MDF and makes it feasible in any physiological pH. Following the logic that substrate channeling can alleviate thermodynamic constraints, we expect that metabolites with positive shadow prices (*i.e*., whose concentration constrains the pathway MDF) will have a higher propensity to be channeled between enzymes, therefore potentially guiding experimental efforts to such locations in search of evidence for substrate channeling. When high throughput methods for identifying channeling are developed, it will be possible to test this hypothesis systematically.

Another solution to this thermodynamic puzzle might be the use of electron acceptors with a higher reduction potential than that of NAD. For example, various organisms operate a malate:quinone oxidoreductase enzyme [75], [76], [77], [78], [79]. In many of these organisms, this enzyme replaces more common NAD-dependent enzymes as the major route of malate oxidation [76], [77], [78], [79]. As shown in Figure 2B, using malate:quinone oxidoreductase enables the TCA cycle to operate at high MDF regardless of the cytoplasmatic pH. The downside of this approach is that less ATP can be produced via oxidative phosphorylation when using a quinone as an electron carrier instead of NAD.

Finally, it is important to note that the TCA cycle is not actually a cycle in many organisms and under various conditions (*e.g.*, [80]). Instead, it often operates in a forked-mode, where malate dehydrogenase catalyzes the favorable direction (*i.e.*, oxaloacetate reduction), eliminating the thermodynamic constraints due to malate oxidation. Remarkably, it was recently suggested that *E. coli* uses a forked TCA cycle even during aerobic growth, despite the low ATP yield associated with this mode [65].

Several natural alternatives to the TCA cycle are also known to support the complete oxidation of organic compounds to CO_2_ [64], [81]. The structures of these pathways are given in Figure S1. Figure 2C compares these metabolic alternatives on the basis of their MDF and ATP yield per glucose. ATP is assumed to be produced from substrate-level phosphorylation and from NAD(P)H through oxidative phosphorylation. The P/O ratio – measuring how many ATP molecules are produced per one oxygen atom being reduced – was taken to be 1.5, the representative value for *E. coli* [82]. Figure 2C suggests that the TCA cycle represents a combination of high ATP yield and high MDF which is better than most of its counterparts – especially if assuming substrate channeling of oxaloacetate (‘TCA channel’) or the usage of quinone instead of NAD (‘TCA MQO’). The oxidative pentose phosphate pathway (‘OxPP’), while producing less ATP molecules than the TCA cycle, supports the highest MDF among all oxidative pathways.

### Substrate-level phosphorylation constrains the Max-min Driving-Force of fermentation pathways

**Figure 3:**
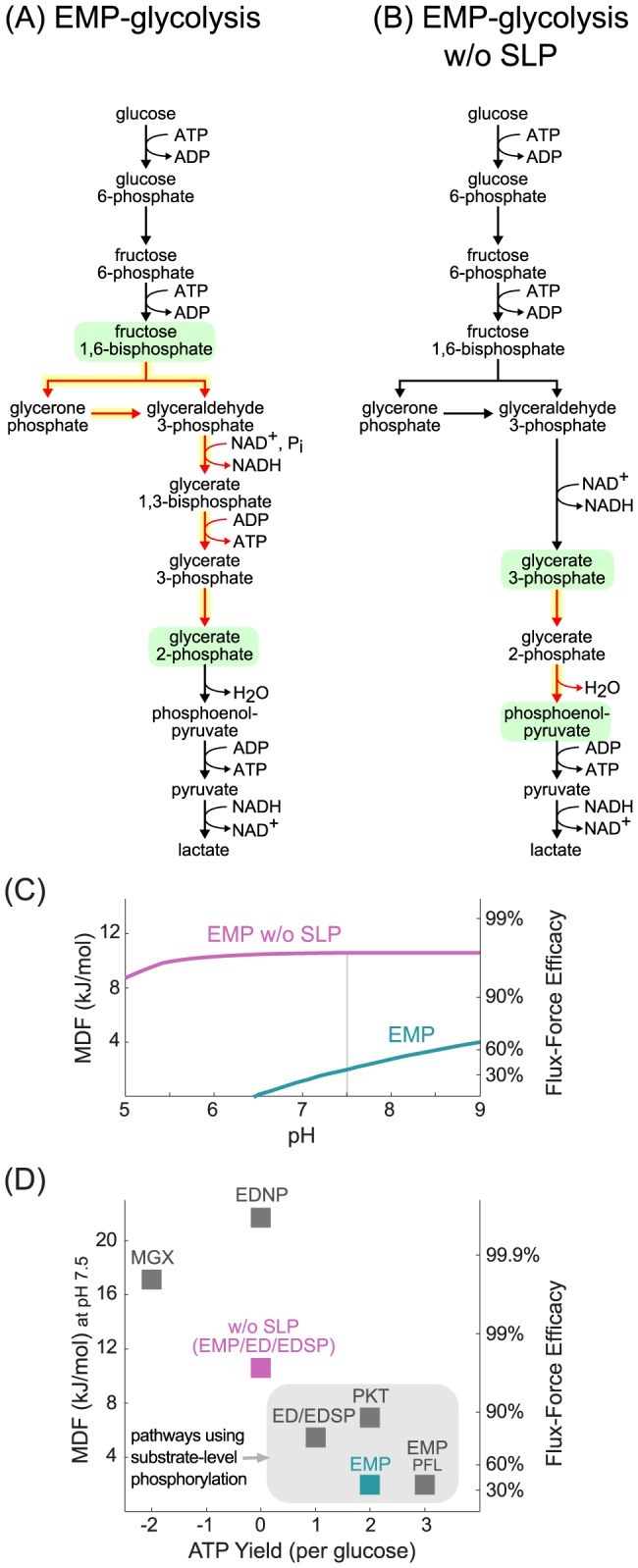
MDF analysis of fermentation pathways. (A) Structure of EMP-glycolysis. (B) Structure of an EMP pathway variant in which substrate-level phosphorylation is bypassed. The reactions marked in red are those with positive shadow prices at pH 7.5. Non-cofactor metabolites shaded in green show positive shadow prices. (C) MDF as function of pH, as calculated for the EMP pathway (cyan) and for an EMP pathway variant in which substrate-level phosphorylation is bypassed (magenta). ‘SLP’ corresponds to substrate-level phosphorylation. The Flux-Force Efficacy axis, on the right, refers to the reactions that dissipate the smallest amount of Gibbs energy, and hence equal to the pathway MDF. The light grey line marks the values corresponding to pH 7.5, the pH used in (D). (D) The MDF and ATP yield per glucose of the different fermentation pathways. ‘ED’ corresponds to the Entner-Doudoroff pathway. ‘EDSP’ represents the semi-phosphorylative ED pathway, known to operate in several hyperthermophilic archaea lineages [84], [88], [89]. ‘EDNP’ represents the non-phosphorylative ED pathway, also known to operate in hyperthermophilic archaea [84], [88], [89]. ‘MGX’ corresponds to a variant of the EMP pathway in which dihydroxyacetone phosphate is converted into the toxic compound methylglyoxal when the concentration of inorganic phosphate becomes limiting [85], [86], [87]. ‘PKT’ represents a pathway, suggested long ago [103], that uses the pentose phosphate pathway in conjunction with the enzyme phosphoketolase that cleaves xylulose-phosphate to glyceraldehyde-phosphate and acetyl-phosphate [90], [91]. ‘EMP PFL’ corresponds to a variant of the EMP pathway that produces more ATP by using the enzyme pyruvate formate lyase and performing substrate-level phosphorylation on of acetyl-phosphate. The structures of all pathways are given in Figure S2.

EMP glycolysis (Figure 3A) is the most investigated fermentation pathway [10]. Substrate-level phosphorylation – coupled to glyceraldehyde 3-phosphate oxidation – is the process responsible for *de novo* ATP synthesis in the pathway (the downstream pyruvate kinase only recoups the ATP invested at the beginning of the pathway) [10]. Nevertheless, some organisms bypass substrate-level phosphorylation altogether such that glyceraldehyde 3-phosphate is directly oxidized to glycerate 3-phosphate, without producing ATP (Figure 3B) [83], [84]. Using the MDF methodology we can offer some insight as to why this may be.

Figure 3C displays the MDF of the EMP pathway – with and without substrate-level phosphorylation – as a function of cellular pH. While the EMP pathway has a very low MDF and seems to be infeasible at pH<6.5, the pathway variant which bypasses substrate-level phosphorylation is characterized by a far higher MDF. This suggests that organisms that do not depend on the degradation of organic compounds for energy conservation – like phototrophs or obligatory respiratory prokaryotes – can profit considerably by skipping substrate-level phosphorylation and operating at a much higher MDF, which can be translated into higher flux or, alternatively, to a lower protein investment required to sustain a given rate [13].

Notably, the trend shown in Figure 3C does not mean that the two substrate-level phosphorylation reactions (glyceraldehyde phosphate dehydrogenase and phosphoglycerate kinase) are the only ones that constrain the pathway MDF. The five reactions marked in red in Figure 3A are those with positive shadow price, showing that multiple reactions constrain the driving force of the EMP pathway. In fact, if any reaction from fructose bisphosphate aldolase to phosphoglycerate mutase had a more favorable Δ_r_G′^o^ value then the MDF of the entire pathway would increase.

Fructose bisphosphate is one of the two non-cofactor metabolites with a positive shadow prices (Figure 3A), and the only one whose concentration upper bound (10 mM) limits the pathway MDF (Methods). Interestingly, the concentration of fructose bisphosphate has been measured to be 15 mM [45], the only glycolytic metabolite whose concentration is higher than 10 mM. This 50% higher concentration adds ∼1 kJ/mol to the driving force of the thermodynamically-constrained reactions, increasing their rather low Flux-Force Efficacies. This example demonstrates how the methodology presented here can be used to rationalize why certain compounds attain higher (or lower) concentrations than others in cells. This further suggests a systematic study of whether an energetic analysis, as the one outlined here, can predict metabolite concentrations on a large scale. However, the measurement of metabolite concentrations using current technologies remains quite noisy, as evident by the dramatic discrepancies between different quantification methods (*e.g.*, [46]). As measurement technology matures, the generality of the connection between the range of metabolite concentrations and the thermodynamically-constrained reactions could be evaluated systematically.

Several glycolytic variants are known to exist in nature and their structures and shown in Figure S2. Figure 3D plots the MDF (at pH 7.5) of each of these pathways against the number of ATP molecules it produces per glucose molecule metabolized. As shown in the figure, there is a clear tradeoff between the MDF and ATP yield, with high- MDF pathways conserving less energy as ATP than pathways with lower MDF. Specifically, the methylglyoxal pathway (‘MGX’) – converting dihydroxyacetone phosphate into the highly reactive compound methylglyoxal [85], [86], [87] – and the non-phosphorylative Entner-Doudoroff (ED) pathway (‘EDNP’) – used by hyperthermophilic archaea [84], [88], [89] – seem to be promising choices for fermenting glucose if ATP production is not important but a high glycolytic flux is required [13].

Within the general trend shown in Figure 3B, some pathways seem better than others. The non-phsophorylative ED pathway, the phosphoketolase pathway (‘PKT’) – using the pentose phosphate pathway and cleaving xylulose-phosphate to glyceraldehyde-phosphate and acetyl-phosphate [90], [91] – and the pyruvate formate lyase pathway (‘EMP PFL’) – cleaving pyruvate to acetyl-CoA and formate and performing substrate-level phosphorylation on acetyl-phosphate – lie on the Pareto front [92], *i.e.*, no other pathway has both a higher MDF and a higher ATP yield. Notably, despite their prevalence in nature, neither the EMP nor the ED pathways are on the Pareto front, which suggests that thermodynamic properties alone are insufficient to explain the structure of central metabolism pathways, as we previously analyzed in detail (*e.g.*, [10]). Specifically, the phosphoketolase and pyruvate formate lyase pathways have higher MDF values than the EMP and ED pathways and yield at least as much ATP. However, it is known that other factors constrain the operation of these pathways in nature. The pyruvate formate lyase enzyme (EC 2.3.1.54) employs an oxygen-sensitive radical mechanism and so can only be used in anaerobic or microaerobic environments [93], [94], [95]. This limitation may explain why the EMP-PFL pathway is less abundant in nature than MDF analysis would lead us to expect.

## Discussion

We introduce a quantitative framework for analyzing the thermodynamic profile of metabolic pathways and identifying reactions that limit metabolic flux within feasible pathways (*i.e.*, require high enzyme levels to sustain a specific rate). While near-equilibrium reactions can significantly increase the protein burden of a pathway, they may have certain advantages. For example, if the direction of a reaction must change quickly in response to some stimulus, operating near equilibrium (and at high enzyme level) is a good strategy: a small change in reactant concentrations can reverse the reaction direction while maintaining a similar absolute flux. This may be particularly important for glycolysis, where some carbon sources require glycolytic flux (*e.g.*, glucose and fructose) and others require flux in the direction of gluconeogenesis (*e.g.*, acetate and succinate). Therefore, fast environmental fluctuations in the availability of carbon sources may require speedy reversal of most glycolytic reactions, which is consistent with recent measurements indicating that reactions in glycolysis mostly operate with low driving-force in *E. coli* [45], [96]. Other functional advantages of working near equilibrium were recently suggested [97], [98].

Our methodology takes into account the physiological conditions, including pH, ionic strength, metabolite concentration ranges and cofactor concentrations. This feature is useful when comparing different organisms hosting the same pathway in different conditions. At the same time, the exact values of some of these parameters are not known with high certainty. In particular, the definition of the metabolite concentration range used in the optimization is challenging, as especially high (>10 mM) and especially low (<1 µM) metabolite concentrations have been measured (*e.g.*, [45]). Furthermore, the physicochemical properties of the metabolites affect their cellular concentrations [44], suggesting that the concentration ranges should be individually tailored to each metabolite.

It is important to remember that the MDF methodology assumes that metabolite concentrations are optimized to achieve the most favorable thermodynamics. These optima are calculated using thermodynamic and stoichiometric data with respect to a single pathway and ignoring the rest of the endogenous metabolic network. Yet, *in-vivo* metabolite concentrations are constrained by many other factors, including their stability, permeability and their participation in other metabolic routes, and so cellular concentrations are unlikely to match these optima precisely. Hence, many of the pathways we analyzed might be more thermodynamically constrained than suggested by the MDF analysis

Our analysis is sensitive to the definition of reactions, *i.e.*, what counts as independent metabolic steps. Merging reactions into a single metabolic step or splitting them into several steps can considerably affect the MDF of a pathway and the Flux-Force Efficacies of its reactions. For example, consider a reaction dissipating 2 kJ/mol and hence operating at a Flux-Force Efficacy of ≈40%. If this reaction is split into two steps, each of these will optimally dissipate 1 kJ/mol and its Flux-Force Efficacy will be only ≈20%. Hence, dividing a pathway into more steps results in lower MDF and Flux-Force Efficacies. Yet, the definition of metabolic steps is not arbitrary. A reaction should be treated as an independent metabolic step if all of its substrates and products are soluble. On the other hand, if two reactions involve a common reactant which remains bound to the enzyme(s), they can be treated as a single metabolic step [9], [74], as was suggested for channeling of oxaloacetate between malate dehydrogenase and citrate synthase.

**Figure 4:**
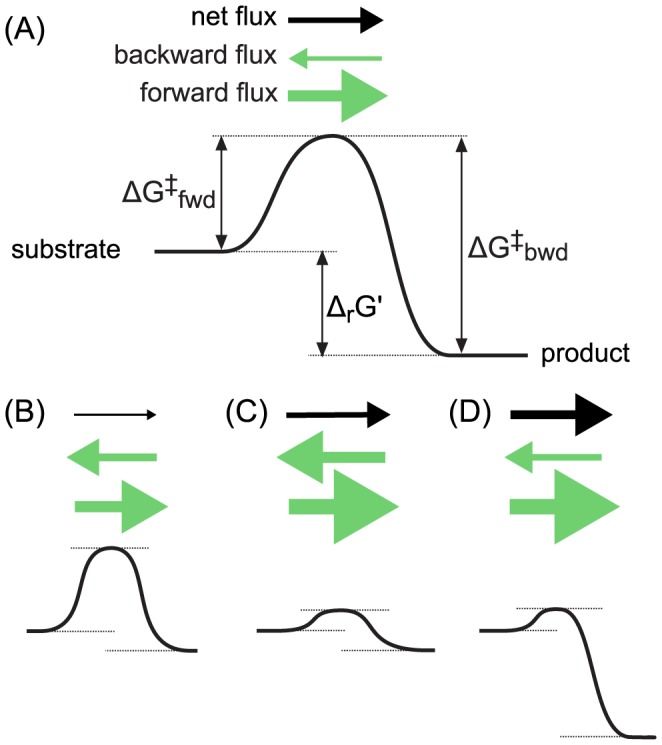
Schematic representation of the interplay between the net reaction flux and the internal and external (i.e., overall or net) energetic profiles. Δ_r_G′ corresponds to the driving force of the net reaction (which depends on the concentrations of the substrates and products); ΔG^‡^_fwd_ to the thermodynamic barrier of the forward reaction, associated with the binding of the substrates and with the different reaction intermediates formed during catalysis; and ΔG^‡^_bwd_ corresponds to the thermodynamic barrier of the backward reaction, associated with the different reaction intermediates formed during catalysis and with the release of the products. All reactions are assumed to be catalyzed by the same amount of enzyme units. (A) High internal thermodynamic barrier and high thermodynamic driving force. (B) High internal thermodynamic barrier and low thermodynamic driving force. (C) Low internal thermodynamic barrier and low thermodynamic driving force. (D) Low internal thermodynamic barrier and high thermodynamic driving force.

Notably, the MDF analysis is insensitive to the kinetic parameters of the enzymes participating in the pathway. In reality, the net reaction flux is determined both by the internal and external reaction energetics. Internal reaction energetics refers to the thermodynamic landscape associated with (i) the binding and release of the reactants from the enzyme's active site; (ii) the different reaction intermediates formed during catalysis; and (iii) the activation energies of converting one reaction intermediate to another [99]. The internal reaction energetics determines the apparent kinetic parameters of the enzyme catalyzing the reaction (*i.e.*, k_cat_, K_M_) [99]. On the other hand, the external reaction energetics refers to the driving force of the net reaction, which depends on the concentrations of the substrates and products, as analyzed in this manuscript. Figure 4 schematically demonstrates the interplay between the net reaction flux and the internal and external energetic profiles. A reaction with a low k_cat_/K_M_ should be compensated by a high driving force (Figure 4A), because otherwise the net flux will be low (Figure 4B). On the other hand, a reaction having a high k_cat_/K_M_ can operate closer to equilibrium (*i.e.*, at a low driving force) and still sustain a high net flux (Figure 4C). Finally, a high driving force and a low internal thermodynamic barrier result in a very high net flux (Figure 4D).

Interestingly, the thermodynamic driving forces of reactions can be directly connected to their control coefficients [27], [30], [100], [101], [102]: for a reaction that has a low thermodynamic driving force, the forward and backward fluxes are considerably larger than the net flux. For a near-equilibrium reaction, then, increasing the enzyme concentration will increase the forward and reverse fluxes to a comparable degree, bringing reactant concentrations even closer to equilibrium. This, in turn, lowers the already-low driving force of the reaction and neutralizes the effect of increasing the enzyme concentration. In brief, increasing the abundance of an enzyme catalyzing a near-equilibrium reaction will have only a modest effect on pathway flux.

On the other hand, a reaction with a high driving force will exert high control on the pathway flux. For such a reaction the net flux roughly equals the forward flux, which is much larger than the reverse flux. In this case, increasing the enzyme abundance will mostly increase the forward flux (in absolute terms). Even if the driving force decreases somewhat, the flux-force efficacy will remain high (see Fig. 1A). Hence, an increase in enzyme abundance will not be compensated and will have a considerable effect on reaction rate. In the Methods and Text S1, we detail the direct mathematical relationship between reaction driving forces and flux control coefficients, which shows that upregulation of enzymes catalyzing reactions with high driving force has a large effect on pathway flux.

Our ultimate goal is to establish a single framework that integrates pathway thermodynamics and enzyme kinetics. We believe it should be possible to reformulate measured kinetic data as thermodynamic potentials and analyze pathways in purely energetic terms. By considering the chemical potential of reaction intermediates and integrating these data with the concentrations of soluble pathway intermediates, one can arrive at a more complete analysis of pathway activity. It remains for future research to develop such an integrated framework.

## Supporting Information

Figure S1 Structure of oxidative pathways. (A) The ubiquitous TCA cycle. (B) The PEP-glyoxylate pathway which was found to operate in *E. coli* under glucose starvation. (C) A pathway used by *Pseudomonas fluorescens* under conditions of aluminum toxicity. (D) The oxidative pentose-phosphate cycle, which can be used to fully oxidize sugars into CO2, thus providing NADPH for cellular activity. The reactions marked in red are those with positive shadow prices (at pH 7.5 and ionic strength of 0.2 M). Non-cofactor metabolites shaded in green show positive shadow prices.
(EPS)

Figure S2 Structure of fermentation pathways. (A) Entner-Meyerhof-Parnas (EMP) glycolysis. Blue arrows correspond to the pyruvate formate lyase shunt. Magenta arrows correspond to a bypass of substrate-level phosphorylation. (B) Entner-Doudoroff (ED) glycolysis. (C) Semi-phosphorylative ED glycolysis, as known to operate in some hyperthermophilic archaea. (D) Non-phosphorylative ED glycolysis, as known to operate in some hyperthermophilic archaea. (E) The methylglyoxal pathway, in which dihydroxyacetone phosphate is converted into the highly reactive compound methylglyoxal when the concentration of inorganic phosphate becomes limiting. (F) The suggested phosphoketolase pathway, which uses the pentose phosphate pathway in conjunction with the enzyme phosphoketolase that cleaves xylulose-phosphate to glyceraldehyde-phosphate and acetyl-phosphate. The reactions marked in red are those with positive shadow prices (at pH 7.5 and ionic strength of 0.2 M). Non-cofactor metabolites shaded in green show positive shadow prices.
(EPS)

Text S1 A mathematical derivation and characteristics of the Max-min Driving Force and its relation to Metabolic Control Analysis.
(PDF)
