# Supplementary material for: Pathway Thermodynamics Highlights Kinetic Obstacles in Central Metabolism
Source: PLoS Comput Biol. 2014 Feb 20;10(2):e1003483. doi: 10.1371/journal.pcbi.1003483 (PMC3930492; doi:10.1371/journal.pcbi.1003483)
Supplement: Text S1 — A mathematical derivation and characteristics of the Max-min Driving Force and its relation to Metabolic Control Analysis. (PDF) [file pcbi.1003483.s003.pdf]

Pathway thermodynamics highlights kinetic obstacles in central metabolism

Text S1

Elad Noor, Arren Bar-Even, Avi Flamholz, Ed Reznik, Wolfram Liebermeister, and Ron Milo

Contents

1 Max-min driving force (MDF) 2
1.1 Original problem . . . 2
1.2 Dual problem . . . 2
1.3 Interpretation – linear pathway example . . . 3

2 Max-min driving force and minimal enzyme cost 4
2.1 Max-min driving force calculation is equivalent to cost minimization of substrate-saturated enzymes . . . 4
2.2 Proof . . . 5

3 Reactions close to equilibrium exert little flux control in simple metabolic pathways 6
3.1 Control coefficients and reaction elasticities in a linear pathway . . . 7
3.2 The elasticities in separable rate laws . . . 7
3.3 Case 1: Substrate-saturated enzymes . . . 8
3.4 Case 2: Enzymes operating in linear range . . . 9
3.5 Consistency with existing formula for flux control coefficients . . . 9
3.6 Summary . . . 10

1 Max-min driving force (MDF)

1.1 Original problem

We formulate the optimization of minimal driving forces as a maximization problem

$$\begin{aligned} \max_{\mathbf{x},B} \quad & B \\ \text{subject to} \quad & \mathbf{S}^\top \mathbf{x} + \mathbf{G}^\circ + \mathbf{1}_{(m)} B \quad \leq \mathbf{0}_{(m)} \\ & \mathbf{x}_{\min} \leq \mathbf{x} \leq \mathbf{x}_{\max} \\ & B \text{ is free} \end{aligned} \tag{1}$$

where $\mathbf{S} \in \mathbb{R}^{n\times m}$; $\mathbf{x}, \mathbf{x}_{\min}, \mathbf{x}_{\max} \in \mathbb{R}^n$; $\mathbf{G}^\circ, \mathbf{0}_{(m)}, \mathbf{1}_{(m)} \in \mathbb{R}^m$; and $B \in \mathbb{R}$. $\mathbf{x}$ represents the natural logarithm of the compound concentrations divided by their standard (1M). $\mathbf{G}^\circ$ represent the standard reaction Gibbs energies (in units of RT). $\mathbf{0}_{(m)}, \mathbf{1}_{(m)}$ are column vectors with only 0 or 1 values, respectively. In order to convert the problem to *standard form*, we define:

$$\mathbf{c} \equiv \begin{pmatrix} \mathbf{0}_{(n)} \\ \mathbf{1} \end{pmatrix} \in \mathbb{R}^{n+1}$$

$$\mathbf{A} \equiv \left(\begin{array}{c|c} \mathbf{S}^\top & \mathbf{1}_{(m)} \\ \mathbf{I}_{(n)} & \mathbf{0}_{(n)} \\ -\mathbf{I}_{(n)} & \mathbf{0}_{(n)} \end{array}\right) \in \mathbb{R}^{(m+2n)\times(n+1)}$$

$$\bar{\mathbf{x}} \equiv \begin{pmatrix} \mathbf{x} \\ B \end{pmatrix} \in \mathbb{R}^{n+1}$$

$$\mathbf{b} \equiv \begin{pmatrix} -\mathbf{G}^\circ \\ \mathbf{x}_{\max} \\ -\mathbf{x}_{\min} \end{pmatrix} \in \mathbb{R}^{m+2n}$$

Then the system in equation 1 is equivalent to the following linear problem:

$$\begin{aligned} \max_{\bar{\mathbf{x}}} \quad & \mathbf{c}^\top \bar{\mathbf{x}} \\ \text{subject to} \quad & \mathbf{A}\bar{\mathbf{x}} \leq \mathbf{b} \\ & \bar{\mathbf{x}} \text{ is free} \end{aligned} \tag{2}$$

1.2 Dual problem

According to duality theory, equation 2 is also equivalent to:

$$\begin{aligned} \min_{\mathbf{y}} \quad & \mathbf{b}^\top \mathbf{y} \\ \text{subject to} \quad & \mathbf{A}^\top \mathbf{y} = \mathbf{c} \\ & \mathbf{y} \geq 0 \end{aligned} \tag{3}$$

which translates to the following system:

$$\begin{aligned} \min_{w,z,u} \quad & -\mathbf{G}^{\circ\top}\mathbf{w} + \mathbf{x}_{\max}^\top \mathbf{z} - \mathbf{x}_{\min}^\top \mathbf{u} \\ \text{subject to} \quad & \mathbf{S}\mathbf{w} + \mathbf{z} - \mathbf{u} = \mathbf{0}_{(n)} \\ & \mathbf{1}_{(m)}^\top \mathbf{w} = 1 \\ & \mathbf{w} \geq \mathbf{0}_{(m)} \\ & \mathbf{z} \geq \mathbf{0}_{(n)} \\ & \mathbf{u} \geq \mathbf{0}_{(n)} \end{aligned} \tag{4}$$

where $\mathbf{w} \in \mathbb{R}^m$, $\mathbf{z}, \mathbf{u} \in \mathbb{R}^n$ and $\mathbf{y} = (\mathbf{w}, \mathbf{z}, \mathbf{u})$.

From the first constraint we get that $\mathbf{u} = \mathbf{S}\mathbf{w} + \mathbf{z}$. Then, substituting $\mathbf{u}$ in the objective yields:

$$-\mathbf{G}^{\circ\top}\mathbf{w} + \mathbf{x}_{\max}^{\top}\mathbf{z} - \mathbf{x}_{\min}^{\top}(\mathbf{S}\mathbf{w} + \mathbf{z}) = -(\mathbf{G}^{\circ\top} + \mathbf{x}_{\min}^{\top}\mathbf{S})\mathbf{w} + (\mathbf{x}_{\max} - \mathbf{x}_{\min})^{\top}\mathbf{z}$$

The right-hand side of this objective is a weighted sum of the different dimensions of $\mathbf{z}$, with positive coefficients (since $\mathbf{x}_{max,j} - \mathbf{x}_{\min,j} > 0$). Also, the only other constraints on $\mathbf{z}$ are the two inequality constraints, $\mathbf{z} \geq \mathbf{0}_{(n)}$ and $\mathbf{S}\mathbf{w} + \mathbf{z} = \mathbf{u} \geq \mathbf{0}_{(n)}$. We can write it in short as: $\mathbf{z} \geq \max(\mathbf{0}_{(n)}, -\mathbf{S}\mathbf{w})$.

Since there are no other constraints and the part of the objective which depends on $\mathbf{z}$ is to minimize $(\mathbf{x}_{\max} - \mathbf{x}_{\min})^{\top}\mathbf{z}$, we get that this inequality is actually and equality:

$$\mathbf{z} = \max(\mathbf{0}_{(n)}, -\mathbf{S}\mathbf{w})$$

And we can also solve for $\mathbf{u}$:

$$\mathbf{u} = \max(\mathbf{S}\mathbf{w}, \mathbf{0}_{(n)})$$

So, we reach this simplified formulation of the dual:

$$\begin{aligned} \min_{\mathbf{w}} \quad & -\mathbf{G}^{\circ\top}\mathbf{w} + \mathbf{x}_{\max}^{\top} \max(\mathbf{0}_{(n)}, -\mathbf{S}\mathbf{w}) - \mathbf{x}_{\min}^{\top} \max(\mathbf{S}\mathbf{w}, \mathbf{0}_{(n)}) \\ \text{subject to} \quad & \textstyle\sum_{i=1}^{m} w_i = 1 \\ & w_i \geq 0 \end{aligned} \tag{5}$$

1.3 Interpretation – linear pathway example

Assume that we have a linear pathway, i.e. $\mathbf{S} \in \mathbb{R}^{m \times (m-1)}$ is a bi-diagonal matrix such as:

$$\begin{pmatrix} -1 & 0 & 0 & 0 & 0 \\ 1 & -1 & 0 & 0 & 0 \\ 0 & 1 & -1 & 0 & 0 \\ 0 & 0 & 1 & -1 & 0 \\ 0 & 0 & 0 & 1 & -1 \\ 0 & 0 & 0 & 0 & 1 \end{pmatrix}$$

and that $x_{min,i}$ and $x_{max,i}$ are the same for all compounds. Note that $-\mathbf{S}\mathbf{w} = \Delta\mathbf{w}$, which is the difference between $\mathbf{w}$ and the same vector shifted by 1 (so that $\Delta w_i = w_i - w_{i-1}$ and we pad the edges with zeros). The dual problem then becomes very simple:

$$\begin{aligned} \min_{w} \quad & f(\mathbf{w}) = \textstyle\sum_i \left[-G_i^{\circ} w_i + x_{\max,i} \max(0, \Delta w_i) - x_{\min,i} \max(-\Delta w_i, 0)\right] \\ \text{subject to} \quad & \textstyle\sum_{i=1}^{m} w_i = 1 \\ & w_i \geq 0 \end{aligned} \tag{6}$$

In the following sections, we will prove that the solution for this problem will always be of the form $\mathbf{w} = (0, \ldots, 0, \phi^{-1}, \ldots, \phi^{-1}, 0, \ldots 0)$, where ϕ is the number of non-zero elements. In other words, $\mathbf{w}$ will contain a single block of non-zero values where all the weights are the same. To show this, we will first prove that any series of consecutive non-zero weights must all have the same value (lemmas 1-2). Then we will conclude that only one such block can exist.

Lemma 1. *Let $\mathbf{w}$ be a non-degenerate feasible optimal solution to the linear problem in (6). Suppose that there is an index i such that the only non-zero values are w_i and w_{i+1}. Then it must be that $w_i = w_{i+1}$.*

x

Proof. We will assume that $w_i \neq w_{i+1}$ and show that this contradicts the optimality of $\mathbf{w}$.

Define $D \equiv \min(w_i, w_{i+1}) > 0$. Now, for any $|\delta| < D$ let the vector $\mathbf{w}$ be changed to $\bar{\mathbf{w}} \equiv \mathbf{w} + \delta(\mathbf{e}_i - \mathbf{e}_{i+1})$, where $\mathbf{e}_i$ is the ith unit vector. This new vector satisfies the constraints of the linear system (all elements are non-negative and sum up to 1).

Importantly, if $\delta < |w_i - w_{i+1}|$, then sign$(\Delta\mathbf{w})$ = sign$(\Delta\bar{\mathbf{w}})$. This means that the objective of the linear problem $f(\mathbf{w} + \delta(\mathbf{e_i} - \mathbf{e_{i+1}}))$ will be a linear function of δ within this range. This fact is a contradiction to the optimality (and non-degeneracy) of $f(\mathbf{w})$, i.e. when $\delta = 0$, since the optimum of a linear function can only be at the boundary of its range. □

Lemma 2. *Let $\mathbf{w}$ be a non-degenerate feasible optimal solution to the linear problem in 6. If there exist $p < q$ where $w_{p-1} = w_{q+1} = 0$ and $\forall i = p, \ldots, q : w_i > 0$ then all these non-zero values are the same, i.e. $w_p = w_{p+1} = \ldots = w_q$.*

Proof. We will again assume that these values are not all the same and then reach a contradiction. Assume that there is $p \leq i \leq q$ such that $w_i \neq w_{i+1}$. Then using the same idea as in the proof of Lemma 1, we can define a vector $\mathbf{d}$ that will be $-x$ for all indexes between p and i and $+y$ for all indexes between $i+1$ and q, and that will sum up to 0. Then, similarly, we can define $\bar{\mathbf{w}} \equiv \mathbf{w} + \delta\mathbf{d}$ and find a small enough δ that will not change the sign of $\Delta\mathbf{w}$. This would mean that $f(\mathbf{w} + \delta\mathbf{d})$ will be linear in δ and this would again lead to a contradiction. □

Conclusion

Lemma 2 means that if $\mathbf{w}$ is a non-degenerate feasible optimal solution then it is a series of "blocks" with zeros between them. Then, using a similar proof to Lemma 2, we can also show that only one such block can exist.

Therefore, for any linear pathway, the solution (or one of the solutions) to the dual problem (Eq. 6) is of the form $\mathbf{w} = (0, \ldots, 0, \phi^{-1}, \ldots, \phi^{-1}, 0, \ldots 0)$, where ϕ is the number of non-zero elements.

Solving the simplified dual for linear pathways

By assuming $\mathbf{w}$ is of the form described in the above conclusion, the only two parameters defining it are the first and last index of the plateau (e.g. p and q). The height is just 1 over the length, i.e. $1/(q-p+1)$. The $x_{\max,q}$ penalty is added only once (for the first index of the plateau) and the $-x_{\min,p+1}$ is added once for the last index. Therefore the dual for linear pathways is equivalent to solving:

$$MDF = \min_{p,q \in \{1\ldots m\}} \left[\frac{-\sum_p^q G_i^\circ + x_{\min,p+1} - x_{\max,q}}{q-p+1} \right]$$

2 Max-min driving force and minimal enzyme cost

2.1 Max-min driving force calculation is equivalent to cost minimization of substrate-saturated enzymes

Consider a linear pathway with N uni-uni reactions, where each product is the substrate of the consecutive reaction. All N enzymes have the exact same k_{cat}^+, are substrate saturated, and are *not* allosterically regulated. The standard Gibbs energies of the reactions (in units of RT) are stored in the vector $\mathbf{G}^\circ \in \mathbb{R}^N$. The log-scale concentrations of the metabolites are stored in the vector $\mathbf{x} \in \mathbb{R}^{N+1}$ satisfying the constraints $\mathbf{x}_{\min} \leq \mathbf{x} \leq \mathbf{x}_{\max}$. The enzyme abundances are given by the vector $\mathbf{E} \in \mathbb{R}^N$.

Now, the vector of reaction Gibbs free energies ($\mathbf{g} \in \mathbb{R}^{N+1}$, also in units of RT) reads $g_i = G_i^\circ + x_{i+1} - x_i$, and according to the second law of thermodynamics, we require that $\mathbf{g} < 0$. Using the separable rate law for

simple MM reactions and assuming full substrate saturation (as in section 2.3.2 in [1], i.e. $[S] \gg K_S$ and $[P] \ll K_P$), the reaction fluxes read $v_i = E_i k^+_{\text{cat}} (1 - \exp(g_i))$. We assume a predefined, stationary flux v_0 through the pathway. To realize this flux, the enzyme abundances must satisfy

$$E_i = \frac{v_0}{k^+_{\text{cat}}} (1 - \exp(g_i))^{-1} \tag{7}$$

and the total enzyme cost will be

$$\mathcal{E} = \sum_i E_i = \frac{v_0}{k^+_{\text{cat}}} \sum_i (1 - \exp(G^\circ_i + x_{i+1} - x_i))^{-1}. \tag{8}$$

One can assume that evolution can adjust the enzyme abundances, and thereby the metabolite levels, in a way that minimizes the total enzyme cost per unit flux. To solve for this, we simply need to minimize $\mathcal{E}$ with respect to c while fixing v_0, k^+_{cat} and G°. This is a convex optimization problem. After dropping the constants v_0 and k^+_{cat}), it can be formulated as:

$$\begin{array}{lll} \min\limits_x & \mathcal{E}(x) = & \sum\limits_i (1 - \exp(g_i))^{-1} \\ \text{where} & \forall i = 1 \dots N & g_i = G^\circ_i + x_{i+1} - x_i < 0 \\ & \forall i = 1 \dots N+1 & \mathbf{x}_{\min} \leq \mathbf{x} \leq \mathbf{x}_{\max}. \end{array} \tag{9}$$

The minimal driving force achieved by the minimal-enzyme cost algorithm can never be better than the value obtained by the MDF algorithm, by definition. This is trivial. However, shortly we will prove that it will also not be worse.

2.2 Proof

Outline of the proof If we optimize for the total enzyme cost, the pathway will contain some stretches of reactions with the following properties:

- The first metabolite hits the upper concentration bound.
- The last metabolite hits the lower concentration bound.
- All reactions in the stretch have the same reaction Gibbs free energy (GFE).
- The reaction before the stretch and the reaction following the stretch have lower reaction GFEs.
- One of these stretches will have the highest GFEs, and this GFE cannot be decreased any further.

Therefore, any minimal enzyme cost solution is a solution of the MDF problem.

Lemma 3. *Let x^* be the solution to equation (9). The following holds $\forall i = 2 \dots N$:*

$$\begin{array}{rcl} x^*_i = x_{\min,i} & \Rightarrow & g^*_{i-1} \geq g^*_i \\ x_{\min,i} < x^*_i < x_{\max,i} & \Rightarrow & g^*_{i-1} = g^*_i \\ x^*_i = x_{\max,i} & \Rightarrow & g^*_{i-1} \leq g^*_i \end{array}$$

Proof. Since x^* is a minimum of $\mathcal{E}$, then $\forall i \in \{2 \dots N\}$ the partial derivative $\frac{\partial \mathcal{E}}{\partial x_i}$ must either be equal to 0, or x_i must be bound such that moving it in the direction of the negative slope is impossible. Explicitly,

$$\begin{array}{rcl} x^*_i = x_{\min,i} & \Rightarrow & \frac{\partial \mathcal{E}}{\partial x_i} \geq 0 \\ x_{\min,i} < x^*_i < x_{\max,i} & \Rightarrow & \frac{\partial \mathcal{E}}{\partial x_i} = 0 \\ x^*_i = x_{\max,i} & \Rightarrow & \frac{\partial \mathcal{E}}{\partial x_i} \leq 0 \end{array}$$

Since x_i affects only g_{i-1} and g_i, the partial derivative of $\mathcal{E}$ with respect x_i is

$$\begin{aligned}\frac{\partial \mathcal{E}}{\partial x_i} &= \frac{\partial (1-\exp(g_{i-1}))^{-1}}{\partial x_i} + \frac{\partial (1-\exp(g_i))^{-1}}{\partial x_i} = \\ &= \frac{\exp(g_{i-1})}{(1-\exp(g_{i-1}))^2} \cdot \frac{\partial g_{i-1}}{\partial x_i} + \frac{\exp(g_i)}{(1-\exp(g_i))^2} \cdot \frac{\partial g_i}{\partial x_i} \\ &= \frac{\exp(g_{i-1})}{(1-\exp(g_{i-1}))^2} - \frac{\exp(g_i)}{(1-\exp(g_i))^2} \ . \end{aligned} \tag{10}$$

Next, we note that the function $h(x) = e^x/(1-e^x)^2$ is monotonically increasing (in the range $x < 0$). Therefore, for any $x, y < 0$ we conclude that $\mathrm{sign}(h(x) - h(y)) = \mathrm{sign}(x - y)$, and in our case

$$\mathrm{sign}\left(\frac{\partial \mathcal{E}}{\partial x_i}\right) = \mathrm{sign}(g_{i-1} - g_i)$$

The conclusion of the lemma follows directly. □

Rest of the proof Using Lemma 3, we can show that the x^* consists of stretches of reactions with equal g_is where the concentration of the first substrate is $x_{\max,q}$ and the concentration of the last product is $x_{\min,p+1}$. If there is a reaction right before or after this stretch, its Gibbs energy is more negative. The sum of all the Gibbs energies of the reactions in this stretch is

$$\sum_{i=q}^{p} g_i^* = \sum_{i=q}^{p} G_i^\circ + x_{\min,p+1} - x_{\max,q} \tag{11}$$

and is a constant. Therefore, there is no way to decrease the Gibbs energy of all the reactions within the stretch simultaneously. Furthermore, since all g_i values are equal, we can calculate it using:

$$\forall i = q \ldots p \ : \ g_i^* = \frac{\sum_{i=q}^{p} G_i^\circ + x_{\min,p+1} - x_{\max,q}}{p - q + 1} \tag{12}$$

and it means that this solution also minimizes the value of the maximal g_i within this stretch of reactions. Therefore, the driving force in the thermodynamic bottleneck reactions, $\min(-g_i)$, will be equal to the MDF.

3 Reactions close to equilibrium exert little flux control in simple metabolic pathways

In this section, we use Metabolic Control Analysis to show a relationship between driving forces and flux control coefficients for a simple but prototypical case. Consider a linear chain of uni-uni reactions in steady state, all supporting the same net flux J. As analyzed in the main article, a reaction with a low driving force is expected to require a high level of its enzyme (assuming all kinetic parameters are similar). Furthermore, the forward and backward fluxes in such a reaction are significantly higher than its net flux J. Thus, if the enzyme level of a low driving force reaction is increased, the forward and backward conversion fluxes will increase almost by the same proportion. Hence, the concentrations of the reactants will further approach their equilibrium ratio and therefore the driving force of the reaction will go even lower. The final outcome is that the potential increase in the net flux is dampened considerably and therefore the effect on J will be minimal. We therefore claim that the reactions with the smallest driving force exert relatively little control over the pathway flux and are thus not expected to be used as regulation points.

3.1 Control coefficients and reaction elasticities in a linear pathway

To show this more rigorously, we use the flux connectivity theorem from Metabolic Control Theory [2]. For the scaled flux control coefficients $C^{\mathrm{J_j}}_{v_i} = \frac{J_i}{J_j}\frac{\partial J_j}{\partial E_i}/\frac{\partial v_i}{\partial E_i} = \frac{E_i}{J_j}\frac{\partial J_j}{\partial E_i}$ between reactions i and flux J_j and the scaled elasticities $\varepsilon^{v_i}_{c_m} = \frac{c_m}{v_i}\frac{\partial v_i}{\partial c_m}$ between metabolite m and reaction i (where c_m is the concentration of m), it reads

$$\sum_i C^{\mathrm{J_j}}_{v_i}\, \varepsilon^{v_i}_{c_m} = 0. \tag{13}$$

Now we consider a linear chain of uni-uni reactions. The first reaction converts metabolite 1 into metabolite 2, the second reaction converts metabolite 2 into metabolite 3, and so on. Since each intermediate metabolite (index i) affects only the previous and the following reaction (with indices i and $i+1$), it has two non-zero elasticities, and the connectivity theorem for the pathway flux J and this metabolite reads

$$C^{\mathrm{J}}_{v_{i-1}}\, \varepsilon^{v_{i-1}}_{c_i} + C^{\mathrm{J}}_{v_i}\, \varepsilon^{v_i}_{c_i} = 0, \tag{14}$$

so

$$\frac{C^{\mathrm{J}}_{v_i}}{C^{\mathrm{J}}_{v_{i-1}}} = -\frac{\varepsilon^{v_{i-1}}_{c_i}}{\varepsilon^{v_i}_{c_i}}. \tag{15}$$

Thus, the ratio of two subsequent flux control coefficients is given by the negative ratio of the reaction elasticities. The same formula also holds for unscaled control and elasticity coefficients.

3.2 The elasticities in separable rate laws

The reversible Michaelis-Menten rate law can be rewritten as a product of three terms [1]:

$$v = V^{+} \cdot \kappa \cdot \gamma \tag{16}$$

where $V^{+} = E\, k^{+}_{\mathrm{cat}}$ is the maximal rate capacity of the enzyme, the binding term κ (ranging between 0 and 1) describes the effects of incomplete substrate saturation, and the thermodynamic term γ (also between 0 and 1) describes the effect of microscopic backward fluxes. The maximal rate capacity V^{+} does not depend on metabolite concentrations. Together with the kinetic term, it yields the microscopic forward rate $v^{+}_i = V^{+}_i \cdot \kappa_i$ of the reaction. The flux-force relationship [3] states that for every reaction, the ratio of forward and backward fluxes is determined by the Gibbs free energy: $v^{+}_i/v^{-}_i = \mathrm{e}^{-g_i}$, and so the ratio between net flux and forward flux reads $v_i/v^{+}_i = (v^{+}_i - v^{-}_i)/v^{+}_i = 1 - e^{g_i}$. This is exactly our thermodynamic term γ_i (see [1]), which is therefore strictly a function of the reaction's Gibbs energy change g_i (in units of RT). Its derivative with respect to g_i reads

$$\frac{\partial \ln \gamma_i}{\partial g_i} = \frac{-e^{g_i}}{1 - e^{g_i}} = -\frac{1}{e^{-g_i} - 1} \tag{17}$$

and the derivative with respect to the logarithmic reactant levels ($x_j \equiv \ln c_j$) can be written as

$$\frac{\partial \ln \gamma_i}{\partial x_j} = \frac{\partial \ln \gamma_i}{\partial g_i} \cdot \frac{\partial g_i}{\partial x_j}. \tag{18}$$

From the definition of g_i we get that

$$\frac{\partial g_i}{\partial x_j} = \frac{G^{\circ}_i + \sum_m n_{mi} x_i}{\partial x_j} = n_{ji} \tag{19}$$

where the n_{ji} are the stoichiometric coefficients for substrates (negative) and products (positive) in reaction i. We can now have a general expression for the scaled elasticities:

$$\varepsilon^{v_i}_{c_j} = \frac{\partial \ln v_i}{\partial x_j} = \frac{\partial \ln V_i^+}{\partial x_j} + \frac{\partial \ln \kappa_i}{\partial x_j} + \frac{\partial \ln \gamma_i}{\partial x_j} = \frac{\partial \ln \kappa_i}{\partial x_j} + n_{ji}\frac{\partial \ln \gamma_i}{\partial g_i}. \tag{20}$$

Taking everything together, the scaled elasticities can therefore be written as

$$\varepsilon^{v_i}_{c_j} = \frac{\partial \ln \kappa_i}{\partial x_j} - \frac{n_{ji}}{e^{-g_r} - 1}. \tag{21}$$

3.3 Case 1: Substrate-saturated enzymes

In this section, we assume that all enzymes in the linear pathway are fully substrate saturated (as in section 2.3.2 in [1], i.e. $[S] \gg K_S$ and $[P] \ll K_P$). In this case, the binding term κ_i for all of the enzymes is insensitive to any of the compound concentrations, and hence:

$$\frac{\partial \ln \kappa_i}{\partial x_j} = 0. \tag{22}$$

Therefore, from Eq.(21) we get that:

$$\varepsilon^{v_i}_{c_j} = -\frac{n_{ji}}{\mathrm{e}^{-g_i} - 1}. \tag{23}$$

For a chain of uni-uni reactions, all stoichiometric coefficients are ± 1, and the flux control coefficients between subsequent reactions $i-1$ and i (and the metabolite c_i in between) show the ratio

$$\frac{C^{\mathrm{J}}_{v_i}}{C^{\mathrm{J}}_{v_{i-1}}} = -\frac{\varepsilon^{v_{i-1}}_{c_i}}{\varepsilon^{v_i}_{c_i}} = \frac{\mathrm{e}^{-g_i} - 1}{\mathrm{e}^{-g_{i-1}} - 1}. \tag{24}$$

Thus

$$\frac{C^{\mathrm{J}}_{v_i}}{C^{\mathrm{J}}_{v_1}} = \frac{\mathrm{e}^{-g_2} - 1}{\mathrm{e}^{-g_1} - 1} \cdot \frac{\mathrm{e}^{-g_3} - 1}{\mathrm{e}^{-g_2} - 1} \cdot \ldots \cdot \frac{\mathrm{e}^{-g_i} - 1}{\mathrm{e}^{-g_{i-1}} - 1} = \frac{\mathrm{e}^{-g_i} - 1}{\mathrm{e}^{-g_1} - 1} \tag{25}$$

so

$$C^{\mathrm{J}}_{v_i} \quad \propto \quad \mathrm{e}^{-g_i} - 1 \tag{26}$$

and, considering the sum theorem, we obtain

$$C^{\mathrm{J}}_{v_i} \quad = \quad \frac{\mathrm{e}^{-g_i} - 1}{\sum_j \mathrm{e}^{-g_j} - 1}. \tag{27}$$

As the driving force of a reaction approaches 0, so does its control coefficient. If a single reaction is completely irreversible (infinite driving force), its control coefficient vanishes. However, if several reactions are completely irreversible, the control coefficients will be undefined because slight changes of enzyme levels – which play a central role in defining the control coefficients – would create a situation in which no steady state is possible.

3.4 Case 2: Enzymes operating in linear range

As a second limiting case, we consider enzymes which have substrate concentrations much lower than the Michaelis-Menten constant, i.e. $x_j \ll K_{M,j}$. In the formulation used in Eq.(16), we can approximate

$$\frac{\partial \ln \kappa_i}{\partial x_j} = \begin{cases} n_{ji} & j \text{ is a substrate of reaction } i \\ 0 & j \text{ is a product of reaction } i \end{cases} . \tag{28}$$

Therefore, Eq. (21) yields

$$\varepsilon^{v_i}_{c_j} = 1 - \frac{n_{ji}}{\mathrm{e}^{-g_i} - 1}, \qquad \varepsilon^{v_i}_{c_{j+1}} = -\frac{n_{(j+1)i}}{\mathrm{e}^{-g_i} - 1}, \tag{29}$$

and for a chain of uni-uni reactions, all stoichiometric coefficients are ± 1, so we obtain

$$\varepsilon^{v_i}_{c_i} = 1 - \frac{-1}{\mathrm{e}^{-g_i} - 1} = \frac{\mathrm{e}^{-g_i}}{\mathrm{e}^{-g_i} - 1}, \qquad \varepsilon^{v_i}_{c_{i+1}} = -\frac{1}{\mathrm{e}^{-g_i} - 1}. \tag{30}$$

The flux control coefficients between subsequent reactions $i-1$ and i (and the metabolite c_i in between) show the ratio

$$\frac{C^{\mathrm{J}}_{v_i}}{C^{\mathrm{J}}_{v_{i-1}}} = -\frac{\varepsilon^{v_{i-1}}_{c_i}}{\varepsilon^{v_i}_{c_i}} = \frac{1}{\mathrm{e}^{-g_{i-1}} - 1} \cdot \frac{\mathrm{e}^{-g_i} - 1}{\mathrm{e}^{-g_i}}. \tag{31}$$

Thus

$$\frac{C^{\mathrm{J}}_{v_i}}{C^{\mathrm{J}}_{v_1}} = \frac{1}{\mathrm{e}^{-g_1} - 1} \cdot \frac{\mathrm{e}^{-g_2} - 1}{\mathrm{e}^{-g_2}} \cdots \frac{1}{\mathrm{e}^{-g_{i-1}} - 1} \cdot \frac{\mathrm{e}^{-g_i} - 1}{\mathrm{e}^{-g_i}} = \frac{e^{-g_1}}{\mathrm{e}^{-g_1} - 1} \cdot (\mathrm{e}^{-g_i} - 1) \cdot \prod_{m=1}^{i} \frac{1}{\mathrm{e}^{-g_m}} \tag{32}$$

so

$$C^{\mathrm{J}}_{v_i} \quad \propto \quad \frac{\mathrm{e}^{-g_i} - 1}{\prod_{m=1}^{i} \mathrm{e}^{-g_m}} \tag{33}$$

The formula shows that driving forces affect the control coefficients in two ways: on the one hand, according to the numerator, strongly driven reactions (large value of $-g_i$) should have a larger control, and reactions with small driving forces ($-g_i$ just above 0) should have little control. On the other hand, the magnitude of the denominator increases along the chain, leading to a tendency for lower control as a reaction is more downstream. In particular, a reaction with large driving force decreases the relative control of all following reactions, not just because the control coefficients must sum to 1, but also because it increases the denominator term for all following reactions (see example in Figure S3).

3.5 Consistency with existing formula for flux control coefficients

The formula (33), for flux control coefficients of enzymes in their linear range, agrees with similar, existing formulae. On the one hand, driving forces g_i and mass-action ratios ρ_i are related by $\rho_i = \mathrm{e}^{-g_i}$. Accordingly, Eq. (33) agrees with Kacser and Burns' formula

$$C^J_1 : C^J_2 : C^J_3 ... \equiv 1 - \rho_1 : \rho_1(1-\rho_2) : \rho_1\, \rho_2(1-\rho_3) \tag{34}$$

from their seminal work on Metabolic Control Analysis [4]. On the other, it is related to a formula from [5] which relates the flux control coefficients to enzyme levels and kinetic constants. Consider again a chain of n reactions (external metabolites concentrations are x_1 and x_{n+1}, internal metabolites are $x_2 \ldots x_n$). As before,

the vector $\mathbf{G}^\circ$ represent the standard reaction Gibbs energies (in units of RT). From the exact flux formula (see [6])

$$J = \frac{[c_1 \prod_{j=1}^{n} e^{-G_j^\circ}] - c_{n+1}}{\sum_{i=1}^{n} \frac{1}{E_i\, k_{-i}} \prod_{m=i+1}^{n} e^{-G_m^\circ}}, \tag{35}$$

follows, by direct differentiation, a formula for the scaled flux control coefficients

$$C_{v_i}^J \propto \frac{1}{E_i\, k_i^-} \prod_{m=i+1}^{n} e^{-G_m^\circ} \tag{36}$$

(compare the equivalent formula in [5]). The absolute values are defined, as usually, by the summation theorem $\sum_i C_{v_i}^J = 1$. This formula depends only on kinetic constants and the enzyme levels, and neither metabolite levels nor driving forces appear explicitly. However, it is equivalent to our formula. We first note that the term $1/(E_i\, k_i^-)$ can be rewritten as c_{i+1}/v_i^-. Then, using that $g_i = G_i^\circ + x_{i+1} - x_i = G_i^\circ + \ln\left(\frac{c_{i+1}}{c_i}\right)$, we rewrite the concentration of the $i+1^{\mathrm{th}}$ metabolite in terms of driving forces:

$$c_{i+1} = c_1 \cdot \prod_{m=1}^{i} \mathrm{e}^{g_m - G_m^\circ} \tag{37}$$

and express the reverse rate v_i^- as $v_i^- = \frac{J}{\mathrm{e}^{-g_i}-1}$. By inserting all this into Eq.(36), we obtain

$$\begin{aligned} C_{v_i}^J \propto \frac{c_{i+1}}{v_i^-} \prod_{m=i+1}^{n} e^{-G_m^\circ} &= \frac{c_1 \cdot \prod_{m=1}^{i} \mathrm{e}^{g_m - G_m^\circ}}{\frac{J}{\mathrm{e}^{-g_i}-1}} \prod_{m=i+1}^{n} e^{-G_m^\circ} \\ &= \frac{c_1}{J} \prod_{m=1}^{n} e^{-G_m^\circ} \frac{\mathrm{e}^{-g_i} - 1}{\prod_{m=1}^{i} \mathrm{e}^{-g_m}} \end{aligned} \tag{38}$$

and, after omitting constant terms, obtain our flux control formula

$$C_{v_i}^J \propto \frac{\mathrm{e}^{-g_i} - 1}{\prod_{m=1}^{i} \mathrm{e}^{-g_m}}. \tag{39}$$

3.6 Summary

In the above two limiting cases, there is a direct relationship between the driving force of a reaction, $-\Delta_r G(i)$, and its control coefficient on the pathway flux, $C_{v_i}^{\mathrm{J}}$. The kinetic parameters of the enzyme, as well as its abundance, do not affect the control coefficient directly, but can still indirectly affect it through changing the driving forces at steady-state. We believe that this trend holds in most kinetic models, i.e., even for non-linear pathways and for cases where some enzyme are neither saturated nor operating in the linear range.

When thinking about the cost of maintaining a certain pathway flux, in terms of enzyme abundances, we would like to focus our analysis on the effect of changing the level of one enzyme. In Metabolic Control Analysis, the sensitivity between an enzyme level and a flux is termed a response coefficient of the enzyme level. Since we constrain ourselves to kinetic models where the rate of each reaction is proportional to the amount of the enzyme catalyzing it, the scaled response coefficients are identical to the scaled control coefficients, $C_{v_i}^{\mathrm{J}}$. Therefore, the response coefficient is higher for reactions that are far from equilibrium and lower for the reactions of minimal driving force. Increasing the enzyme level in such a reaction will have a weaker effect on the pathway flux than increasing the level of an enzyme with a high driving force.

References

1. Noor E, Flamholz A, Liebermeister W, Bar-Even A, Milo R (2013) A Note on the Kinetics of Enzyme Action: a Decomposition that Highlights Thermodynamic Effects. FEBS letters .

2. Heinrich R, Schuster S (1996) The Regulation of Cellular Systems. Chapman & Hall.

3. Beard DA, Qian H (2007) Relationship between thermodynamic driving force and one-way fluxes in reversible processes. PloS one 2: e144.

4. Kacser H, Burns J (1973) The control of flux. Symp Soc Exp Biol 27: 65-104.

5. Heinrich R, Klipp E (1996) Control analysis of unbranched enzymatic chains in states of maximal activity. J Theor Biol 182: 243-252.

6. Klipp E, Heinrich R (1999) Competition for enzymes in metabolic pathways: implications for optimal distributions of enzyme concentrations and for the distribution of flux control. BioSystems 54: 1-14.
